# PCDHGA9 represses epithelial-mesenchymal transition and metastatic potential in gastric cancer cells by reducing β-catenin transcriptional activity

**DOI:** 10.1038/s41419-020-2398-z

**Published:** 2020-03-30

**Authors:** Junyong Weng, Shanbao Li, Hao lin, Haitao Mei, Yang Liu, Chao Xiao, Zhonglin Zhu, Weiwei Cai, Xusheng Ding, Yushuai Mi, Yugang Wen

**Affiliations:** 1 Department of General Surgery, Shanghai General Hospital, School of Medicine, Shanghai Jiaotong University, 200080 Shanghai, China; 2 Department of Gastrointestinal Surgery, Changzheng Hospital, Second Military Medical University, 200003 Shanghai, China; 3 Department of General Surgery, Jiading District Central Hospital Affiliated Shanghai University of Medicine & Health Sciences, 201800 Shanghai, China; 4 Department of Medicine II, University Hospital, Liver Centre Munich, LMU, Munich, 80539 Germany; 5 Department of General Surgery, Shanghai Huashan Hospital, Fudan University, 200000 Shanghai, China; 6 Department of General Surgery, Henan Provincial People’s Hospital, 450003 Zhengzhou, Henan China; 7 Department of Medicine, The Third Hospital of Quanzhou, 362000 Quanzhou, China; 8 Department of General Surgery, The Second Hospital of Shandong University, 250033 Jinan, Shandong China

**Keywords:** Gastric cancer, Metastasis

## Abstract

Gastric cancer (GC) has a high mortality rate, and metastasis is the main reason for treatment failure. It is important to study the mechanism of tumour invasion and metastasis based on the regulation of key genes. In a previous study comparing the expression differences between GES-1 and SGC-7901 cells, PCDHGA9 was selected for further research. In vitro and in vivo experiments showed that PCDHGA9 inhibited invasion and metastasis. A cluster analysis suggested that PCDHGA9 inhibited epithelial-mesenchymal transition (EMT) through the Wnt/β-catenin and TGF-β pathways. Laser confocal techniques and western blotting revealed that PCDHGA9 inhibited the nuclear translocation of β-catenin, regulated T cell factor (TCF)/ /lymphoid enhancer factor (LEF) transcriptional activity, directly impacted the signal transmission of the TGF-β/Smad2/3 pathway, strengthened the adhesion complex, weakened the effects of TGF-β, and blocked the activation of the Wnt pathway. In addition, PCDHGA9 expression was regulated by methylation, which was closely related to poor clinical prognosis. The aim of this study was to elucidate the molecular mechanism by which PCDHGA9 inhibits EMT and metastasis in GC to provide a new theoretical basis for identifying GC metastasis and a new target for improving the outcome of metastatic GC.

## Introduction

Gastric cancer (GC) was the second leading cause of cancer-related death and the sixth most frequently diagnosed cancer worldwide in 2018^1^. With its poor prognosis, overall 5-year survival rate of GC is still less than 30%, and distant metastasis is the major barrier to improve the therapeutic effect^2,3^. Tumour metastasis is a multistep and multi-molecular process^4^; therefore, a thorough understanding of the mechanism underlying GC metastasis is significant for developing innovative therapeutic tactics.

Epithelial-mesenchymal transition (EMT) is essential in the initial stages of GC metastasis; in this process, the epithelial cell cytoskeleton is reorganized, and tight junctions are dissolved^5^. Importantly, epithelial cells undergo a developmental switch that enables them to acquire mesenchymal characteristics, resulting in a decrease in adhesion and cell polarity and an increase in motility and invasiveness during EMT^6^. This process is also associated with upregulation of N-cadherin, Vimentin and Slug and concomitant downregulation of E-cadherin^7^. EMT involves complex mechanisms regulated by many signalling pathways, including the Wnt/β-catenin pathway, the transforming growth factor-β (TGF-β)/Smad2/3 pathway and other pathways^8,9^. Accumulating evidence indicates that the canonical Wnt pathway negatively regulates E-cadherin and induces EMT by protecting the significant factor β-catenin from proteasome degradation^10,11^. Normally, β-catenin interacts with cadherin and forms a complex at the membrane. TGF-β may disassociate this complex to release β-catenin, which can subsequently translocate to the nucleus; this is required for posttranscriptional regulation of β-catenin and activation of EMT^12^.

According to some models, downregulation of cadherin leads to a reduction in β-catenin membrane binding, mediating its effect on gene transcription^13,14^. As members of the cadherin family, protocadherins (PCDHs) likely play critical roles in the establishment and function of specific cell–cell connections in the brain^15^. However, little information is available about the relationship between PCDHs and either tumorigenesis or nuclear signals. Our previous study indicates that PCDHGA9 may serve as a potential novel biomarker in GC and is closely associated with GC patient outcomes^16^. Nevertheless, we have not determined how PCDHGA9 is downregulated in GC. It is well known that the occurrence and development of GC are characterized by the gradual formation of multiple epigenetic and genetic mutations. DNA methylation could cause promoter hypermethylation and specific gene inactivation^17–19^. Here, we assessed the methylation and inactivation frequency of PCDHGA9 in cancer tissues and investigated its functions in the progression of GC. In our previous study, we clearly demonstrated that PCDHGA9 suppresses GC cell proliferation via the Wnt/β-catenin pathway and inhibits EMT by suppressing TGF-β/Smad2/3 pathway activation. Importantly, we analysed cDNA array information via Ingenuity Pathway Analysis (IPA) and found that there might be a connection between the Wnt/β-catenin and TGF-β/Smad2/3 pathways in EMT signalling. In the present study, we further determined that PCDHGA9 could directly interact with β-catenin to form a complex at the GC cell membrane to inhibit EMT, and we provide evidence of the association between the canonical Wnt pathway and the TGF-β pathway.

In this study, we demonstrated that PCDHGA9 is downregulated in GC tissues, especially in metastatic GC. Moreover, we found that the loss of PCDHGA9 results in the nuclear translocation of β-catenin and the promotion of EMT in GC cells, leading to enhanced metastatic and invasive abilities. Furthermore, we revealed a negative correlation between PCDHGA9 and N-cadherin, Vimentin and Twist and a positive correlation between PCDHGA9 and E-cadherin expression in GC specimens. In addition, we showed that PCDHGA9 interacts with β-catenin to antagonize the canonical Wnt pathway and inhibit the TGF-β/Smad2/3 pathway. Importantly, promoter hypermethylation was correlated with PCDHGA9 downregulation and poor prognosis in GC patients. Taken together, our data show that PCDHGA9 is a novel Wnt/β-catenin signalling pathway suppressor and a potential therapeutic target in metastatic GC.

## Methods

### Patients and ethical approval

A total of 66 pairs of fresh frozen GC and normal tissue specimens were obtained in accordance with the guidelines approved by the institutional review boards of Shanghai General Hospital, Shanghai Jiao Tong University School of Medicine. None of the patients received chemotherapy, radiotherapy or other related antitumour therapies before surgery. The final patient follow-up date was 1 August 2009, and the median observation time for the patients was 37.5 months (range, 9–83 months). At least two certified pathologists confirmed all diagnoses, and the tumour stage and grade classification were based on pathological findings according to International Union Against Cancer guidelines. This study was conducted in accordance with the World Medical Association Declaration of Helsinki. All patients signed informed consent forms. The clinicopathological characteristics of the patients are summarized in Table 1. Another group of 75 matched pairs of GC and normal mucosa samples was used for the tissue microarray (TMA), which was obtained from Shanghai Superchip Biotechnology Company.

**Table 1:** Association between PCDHGA9 expression and clinicopathologic features in 66 gastric cancer patients.

Characteristics	No	Expression		*p*-value
		High	Low	
Age (years)				0.586
<65	34	15	19	
≥65	32	14	18	
Sex				0.246
Female	27	10	17	
Male	39	19	20	
Location				0.906
Gastric fundus	3	1	2	
Gastric corpus	28	12	16	
Pylorus	35	16	19	
Tumour size (cm)				0.011
<3	17	12	5	
≥3	49	17	32	
UICC stage				<0.001
I + II	25	24	1	
III + IV	41	5	36	
Lymph node metastasis				<0.001
No	16	15	1	
Yes	50	14	36	
Differentiation				<0.001
High + Moderate	17	16	1	
Low	49	13	36	
Methylation				0.033
Methylated	30	9	21	
Unmethylated	36	20	16	

### Quantitative real-time polymerase chain reaction (qRT-PCR)

Total RNA from cell lines and matched primary and normal mucosa tissues from GC patients was extracted using TRIzol reagent (Invitrogen, California, USA). cDNA was reverse transcribed from 2 μg of total RNA using a HiScript qRT SuperMix for qPCR kit (Vazyme, Nanjing, China) according to the manufacturer’s instructions. qRT-PCR was performed using a ChamQ SYBR qPCR Master Mix kit (Vazyme, Nanjing, China). The primers used are listed in Table 2. GAPDH was used as an internal control. The mRNA levels were calculated using the 2^−^^(ΔCt sample–△ΔCt control)^ method, and all experiments were run in triplicate.

**Table 2:** Sequences of primers for quantitative reverse transcription-PCR.

Gene	Forward primer (5′–3′)	Reverse primer (5′–3′)
PCDHGA9	GCTCATTTCGGTGGAAGAT	CACTGGGCTAAACAGAGAT
E-cadherin	AGGCCAAGCAGCAGTACATT	CATTCACATCCAGCACATCC
Slug	TCTTCACTCCGAAGCCAAAT	TCTGTGGGTGTGTGTGTGTG
Vimentin	CGAAACTTCTCAGCATCACG	GCAGAAAGGCACTTGAAAGC
N-cadherin	TTTGAGGGCACATGCAGTAG	ACTGTCCCATTCCAAACCTG
Twist	GCCAGGTACATCGACTTCCTCT	TCCATCCTCCAGACCGAGAAGG
GAPDH	GGGAAGGTGAAGGTCGGAGT	GGGGTCATTGATGGCAACA

### Western blot analysis

Total protein or nuclear protein was extracted according to the manufacturer’s instructions (Yeasen, Shanghai, China). Protein concentrations were measured using a BCA assay (Beyotime Biotechnology, Jiangsu, China). Equal amounts of protein (50 μg) were separated on SDS-PAGE gels and transferred onto polyvinylidene difluoride membranes (Millipore, Billerica, USA). The membranes were first blocked for nonspecific binding in 5% skim milk in TBST buffer at room temperature for 1.5 h, incubated with specific primary antibodies at 4 °C overnight, and incubated with a horseradish peroxidase-conjugated secondary antibody at room temperature for 1 h. The bound antibody was detected with ECL reagent (MultiSciences, Hangzhou, China). The primary antibodies for western blotting are listed in Table 3.

**Table 3:** Primary antibodies for western blotting.

Antibody	Concentration	Specificity	Company
PCDHGA9	1:500	Mouse polyclonal	Abcam
E-cadherin	1:800	Rabbit monoclonal	Cell Signaling Technology
Slug	1:2000	Mouse monoclonal	Abcam
Vimentin	1:800	Rabbit monoclonal	Cell Signaling Technology
N-cadherin	1:1000	Mouse monoclonal	Abcam
β-catenin	1:1000	Rabbit monoclonal	Abcam
c-Myc	1:1000	Rabbit monoclonal	Cell Signaling Technology
Cyclin D1	1:1000	Rabbit monoclonal	Cell Signaling Technology
Survivin	1:1000	Rabbit monoclonal	Cell Signaling Technology
MMP7	1:500	Rabbit monoclonal	Santa Cruz
GAPDH	1:1000	Mouse monoclonal	Santa Cruz
Twist	1:1000	Rabbit monoclonal	Cell Signaling Technology
Histone H3	1:2000	Rabbit monoclonal	Cell Signaling Technology
ATP1A1	1:1000	Rabbit monoclonal	Cell Signaling Technology
Flag	1:1000	Rabbit monoclonal	Cell Signaling Technology
His	1:1000	Rabbit monoclonal	Cell Signaling Technology

### Cells, primers, plasmids and shRNAs

The human GC cell lines SGC-7901, MGC-803, MKN-45, MKN-28, HGC-27, AGS, BGC-823 and GES-1 were obtained from the Type Culture Collection of the Chinese Academy of Sciences (Shanghai, China). The bisulfite sequencing PCR (BSP) primers were as follows: PCDHGA9-1, forward 5′-GTTATGGGAGGTTAGGGTTAG-3′ and reverse 5′-AATCAATAACTTCTAAAAATAAATAAC-3′; and PCDHGA9-2, forward 5′-GTTATTTATTTTTAGAAGTTATTGATT-3′ and reverse 5′-CAAAATTTATATACCACTTTTCC-3′. The pLVX-IRES-puro-3×Flag-hPCDHGA9 and pHis-CMV4-hβ-catenin plasmids were used to ectopically express PCDHGA9 and β-catenin, respectively. The shRNA target sequences were as follows: sh-PCDHGA9-1, GCGGAAGATTAGTCCTGCTAT; sh-PCDHGA9-2, CCCAAATTCTTGACCGAGAAA and sh-PCDHGA9-3, CCTGCAAGTGACTGACATCAA.

### Scratch wound healing assay

MKN-28, MKN-45, SGC-7901 and MGC-803 cells with stable plasmid transfection were seeded in 6-well plates. After the cells reached confluence, a linear uniform scratch was generated with a standard 10-μl pipette tip at the centre of the well. Photographs were captured at the indicated time intervals in the different cell lines, and wound widths were quantified and compared to baseline values in experiments that were repeated three times. A two-tailed unpaired *t*-test was used to analyse the time-course data.

### Transwell cell migration and invasion assays

Each group of cells (2 × 10^4^/chamber) was seeded in 200 μl of serum-free media in the upper chamber of an 8.0-μm pore transwell (Corning, New York, USA) without (migration) or with (invasion) Matrigel (BD Bioscience, USA). The lower chamber contained 600 μl of medium supplemented with 10% FBS as a chemokine. After 24 h of incubation, the cells that migrated or invaded to the membrane surface exposed to the lower chamber were fixed with methyl alcohol, stained with a 0.1% crystal violet solution and then counted in 10 random fields. The experiments were repeated in triplicate.

### In vivo tumour xenograft and metastasis experiments

For the subcutaneous tumour growth assay, 150 μl of 2 × 10^6^ cells were injected subcutaneously into the groins of nude mice (*n* = 6 for each group). Tumour diameter was measured every 5 days. One month later, the mice were sacrificed, and tumours were harvested. The tumour volumes were calculated using the formula (volume = length × width^2^ × 1/2).

For the peritoneal metastatic model, 1 × 10^6^ stable cells were injected into the abdominal cavities of nude mice (*n* = 6 per group). After 1 month, all the mice were sacrificed, and the number of peritoneal metastatic nodules was counted. Fresh subcutaneous tumour and metastatic nodule tissues were removed and stored at −80 °C.

For the hepatic metastasis assay, mice were anaesthetized, and a lateral incision was made in the abdomen. Fifty microliters containing 1 × 10^6^ cells stably transduced with plasmid were injected into the spleen. Then, the abdomen was closed (*n* = 6 per group). Aseptic techniques were strictly followed during the entire operation. Six weeks later, all the mice were sacrificed, and the livers were harvested and fixed with 4% paraformaldehyde for H&E staining. Then, the number of metastatic nodules was counted.

Animal experiments were conducted with 4-week-old male BALB/c nude mice (Institute of Zoology, Chinese Academy of Sciences, Shanghai, China). All animal assays were approved by the Institutional Animal Care and Use Committee of Shanghai General Hospital.

### Immunofluorescence assay

Cells were seeded onto sterilized coverslips, incubated for 48 h, washed once with PBS, fixed in 4% paraformaldehyde for 20 min at room temperature, washed three times with PBS and permeabilized with 0.1% Triton X-100 for 3 min. Then, the cells were washed three times with PBS and blocked for 1 h with blocking buffer (1% bovine serum albumin in PBS buffer, pH 7.4).

The coverslips were incubated overnight with an antibody against β-catenin (1:100), followed by an additional 2 h incubation with a fluorescently labelled secondary antibody (Abcam, London, UK) in PBS. Cell nuclei were stained with 4′,6 diamidino-2-phenylindole (DAPI) for 1 min, and the slides were viewed under a confocal laser-scanning microscope (Leica, Wetzlar, Germany).

### Immunoprecipitation

Total protein from 293 T cells was extracted using RIPA lysis buffer. Cell lysates were immunoprecipitated with the indicated antibodies conjugated to protein A/G beads (Santa Cruz Biotechnology) overnight. Then, the beads were washed and boiled in SDS loading buffer. Using western blotting, the immunoprecipitated protein complexes were detected.

### TOP/FOP flash assay

The indicated cells were seeded in 24-well plates at 2 × 10^4^ cells per well. After 24 h, the TOP flash or negative control FOP reporter plasmid (Millipore, Billerica, MA, USA) and a Renilla luciferase plasmid were introduced into these cells. Twenty-four hours later, the luciferase activity was measured with a dual-luciferase reporter kit (Promega, Madison, WI, USA).

### Chemicals

XAV-939 and CHIR-99021 were obtained from MedChemExpress (New Jersey, USA). TGF-β1 was purchased from Peprotech (New Jersey, USA). 5-Aza was obtained from Sigma-Aldrich (Missouri, USA).

### Statistical analysis

Data were analyzed using one-way analysis of variance or Student’s t-test for comparison between groups. The associations between PCDHGA9 expression and other clinicopathological characteristics were analysed by χ^2^ test. The correlations between PCDHGA9 and E-cadherin, N-cadherin, Vimentin or Twist were determined using the Spearman rank correlation. Metastasis-free survival (MFS) in patients was analysed by the Kaplan-Meier method with a log-rank test. All statistical analyses were carried out using the SPSS 22.0 statistical software package (SPSS Inc., Chicago, IL, USA). A *p*-value <0.05 indicated a significant difference.

## Results

### PCDHGA9 is downregulated and significantly suppresses proliferation and wound healing abilities in GC cells

**Fig. 1:**
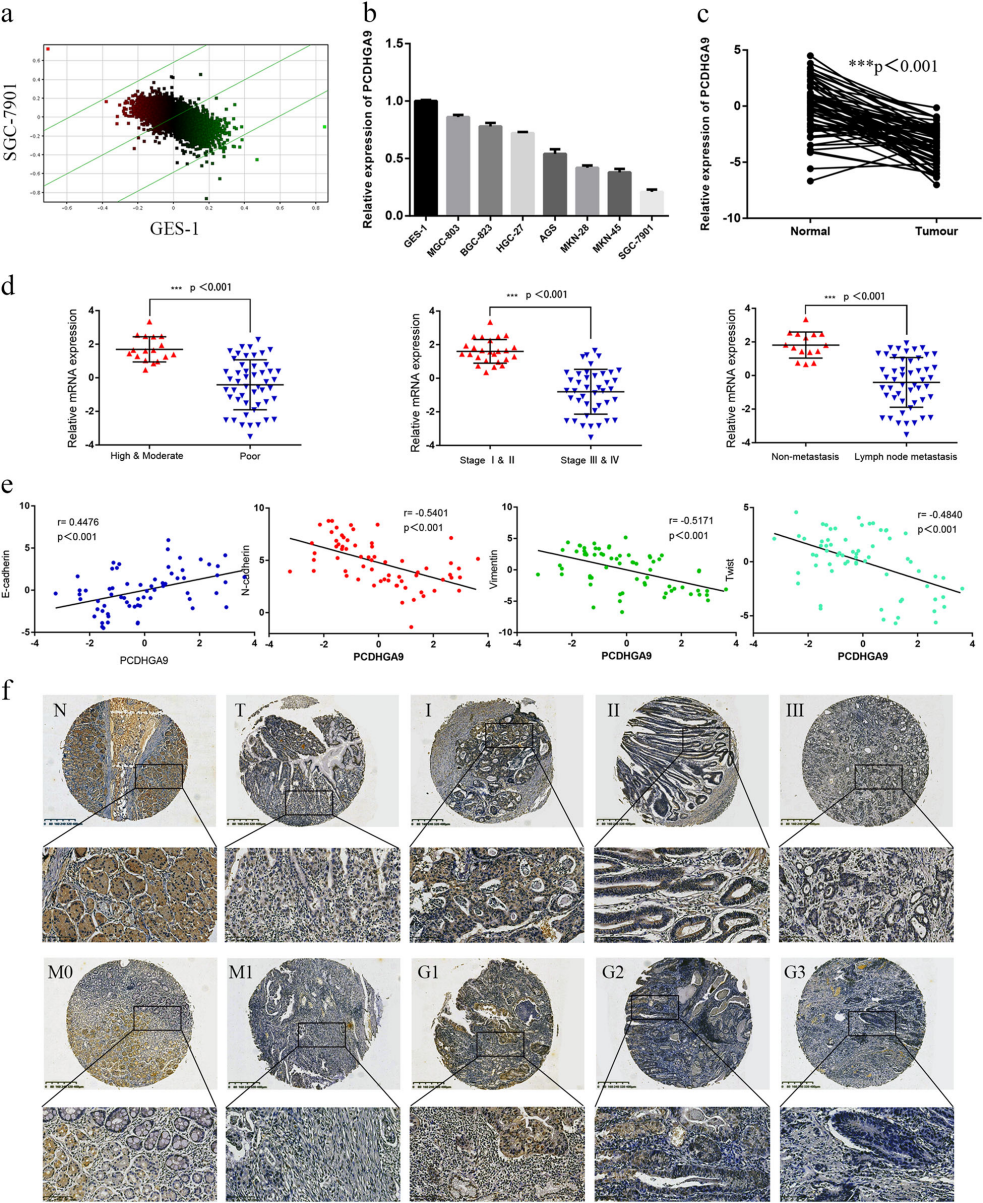
PCDHGA9 is downregulated in GC and correlated with the Clinical pathologic characteristics. PCDHGA9 expression in GC tissue. **a** Scatter diagram of the different gene expression patterns between SGC-7901 and GES-1 cells. **b** The relative mRNA expression of PCDHGA9 was downregulated in GC cell lines but highly expressed in GES-1 cells. **c** The mRNA level of PCDHGA9 in GC tissues was significantly lower than that in matched normal gastric tissues. **d** The relative expression of PCDHGA9 in GC tissues stratified by differentiation status was compared by qPCR. Relative PCDHGA9 expressions in early stage (I and II) and nonmetastatic patients were much higher than in advanced stage (III and IV) and metastatic tumour tissues. **e** Spearman’s correlation analyses showed that PCDHGA9 was positively correlated with E-cadherin expression but negatively associated with N-cadherin, Vimentin and Twist expression in tissues from 66 GC patients (E-cadherin: r = 0.4476, *p* < 0.001; N-cadherin: r = −0.5401, *p* < 0.001; Vimentin: r = 0.-0.5171, *p* < 0.001; Twist: r = −0.4840, *p* < 0.001). **f** Representative images of PCDHGA9 expression in normal gastric mucosa and GC tissues with from patients with stage I, stage II, stage III, M0, M1, G1, G2 and G3 status. All data are presented as the mean ± SEM. ***p < 0.001 by two-tailed Student’s *t*-test.

A cDNA microarray was used to assess differentially expressed genes between the normal gastric mucosa cell line GES-1 and the highly invasive GC cell line SGC-7901 (Fig. 1a). We found that PCDHGA9, ADAMTS12, KRT77, BDP1 and PLVAP were up or downregulated compared to their respective expression in normal cells and might be associated with malignant biological behaviours in GC. We then blocked the expression of these genes in the SGC-7901 cell line with targeted short hairpin RNAs (shRNAs) and observed the wound healing ability of the cells. The results showed that silencing PCDHGA9, ADAMTS12, KRT77 or BDP1, PLVAP promoted or inhibited the migration of GC cells compared to the control conditions (Sup Fig. 1a). In addition, the loss of PCDHGA9, ADAMTS12, KRT77 or BDP1, PLVAP accelerated or suppressed SGC-7901 cell proliferation and growth as determined by a Celigo image cytometer (Sup Fig. 1b, c). Enhanced N-cadherin and Vimentin and decreased E-cadherin expression were observed in cells with PCDHGA9, ADAMTS12 or KRT77 knockdown. By contrast, cells with BDP1 or PLVAP knockdown showed the opposite results (Sup Fig. 1d). Importantly, we found that sh-PCDHGA9 could remarkably alter the proliferation and migration abilities of GC cells more than other genes and might thus play an important role in advanced GC.

### Downregulation of PCDHGA9 is associated with EMT-related proteins in GC

In this study, we examined PCDHGA9 expression in GC cell lines and 66 matched pairs of fresh frozen GC and normal adjacent tissues with qRT-PCR. The results showed that PCDHGA9 expression was significantly reduced in GC cell lines and GC tissues (Fig. 1b, c). We then compared PCDHGA9 expression in GC tissues with varying levels of differentiation and found that, consistent with IHC results in another group of 75 matched pairs of GC and normal tissues (Fig. 1f), PCDHGA9 was more highly expressed in the groups with high or moderate differentiation than in the group with poor differentiation. Moreover, higher PCDHGA9 expression was observed in patients with stages I and II disease than in patients with stages III and IV disease. More importantly, we found that PCDHGA9 was significantly upregulated in patients without metastasis (Fig. 1d–f). PCDHGA9 expression in GC tissues was negatively correlated with differentiation, tumour size, lymph node metastasis, UICC stage and methylation level (Table 1). Taken together, these results suggest that PCDHGA9 is not only engaged in EMT but also correlated with the clinicopathological characteristics of GC. In general, PCDHGA9 acts as a tumour suppressor, and low PCDHGA9 expression indicates an aggressive type in GC.

Next, we examined the expression levels of EMT-related markers (E-cadherin, Vimentin, N-cadherin and Twist) and their association with PCDHGA9 expression in the above 66 GC specimens via qRT-PCR. Low PCDHGA9 expression was frequently correlated with low E-cadherin expression and high N-cadherin, Vimentin and Twist expression in tumour tissues (Fig. 1e). Pearson’s correlation analyses based on qRT-PCR revealed a positive correlation between PCDHGA9 and E-cadherin expression (r = 0.4476, *p* < 0.001) but negative associations between PCDHGA9 and N-cadherin (r = −0.5401, *p* < 0.001), Vimentin (r = −0.5171, *p* < 0.001) and Twist (r = −0.4840, *p* < 0.001) expression. Taken together, these results suggest that PCDHGA9 is not only correlated with the clinicopathological characteristics of GC but also involved in EMT.

### PCDHGA9 suppresses the migration and invasion abilities of GC cells in vitro

**Fig. 2:**
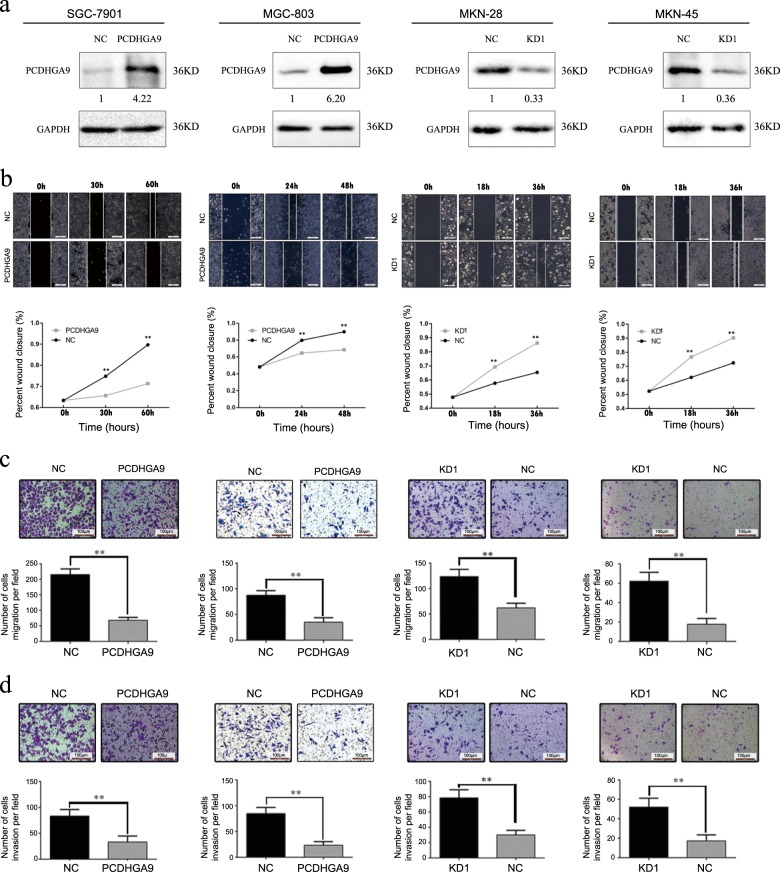
PCDHGA9 inhibits the migration and invasion abilities of GC cells in vitro. **a** SGC-7901 and MGC-803 cells and MKN-28 and MKN-45 cells transfected with PCDHGA9 overexpression or silencing vectors, respectively, were verified by western blotting. GAPDH was used as an internal control. The effects of PCDHGA9 overexpression or depletion on the **b** wound healing, **c** migration and **d** invasion abilities of the indicated cell lines. Original magnification: ×100. NC group infected with negative control lentivirus, KD-1 group infected with lenti-shRNA-1. The data are presented as the mean ± SEM of three independent experiments. **p < 0.01 by two-tailed Student’s *t*-test.

To explore the effect of PCDHGA9 on EMT in GC cells, we assessed E-cadherin and Vimentin expression in different GC cell lines (Sup Fig. 3a). MKN-28 and MKN-45 cells manifested the highest levels of E-cadherin expression and lowest levels of Vimentin expression and were thus chosen for PCDHGA9 silencing experiments. By contrast, SGC-7901 and MGC-803 cells showed the opposite results and were thus selected for PCDHGA9 overexpression experiments (Sup Fig. 3b). We transfected lentiviral vector containing shRNA targeting PCDHGA9 (lenti-shRNA) into GC cells. Compared with the negative control shRNA group, the lenti-shRNA1 (KD-1) showed obviously silenced PCDHGA9 expression whereas the other two shRNAs (KD-2 and KD-3) showed moderate decreases (Sup Fig. 3c). Therefore, KD-1 was chosen for subsequent experiments. To explore the potential functions of PCDHGA9, we established cell with stable PCDHGA9 overexpression (SGC-7901-PCDHGA9 and MGC-803-PCDHGA9) and knockdown (MKN-28- KD-1 and MKN-45-KD-1) (Fig. 2a).

Next, we investigated the metastatic ability of GC cells with different expression levels of PCDHGA9. The wound healing assays suggested that cells with higher PCDHGA9 expression demonstrated significantly slower wound closure than their respective control cells (Fig. 2b). These results were confirmed by transwell migration assays (Fig. 2c). Furthermore, transwell invasion assays showed that PCDHGA9 overexpression or knockdown resulted in lower or higher invasion rates, respectively (Fig. 2d). Thus, we conclude that PCDHGA9 overexpression inhibits the migration and invasion of GC cells in vitro.

### PCDHGA9 modulates EMT in GC cells

**Fig. 3:**
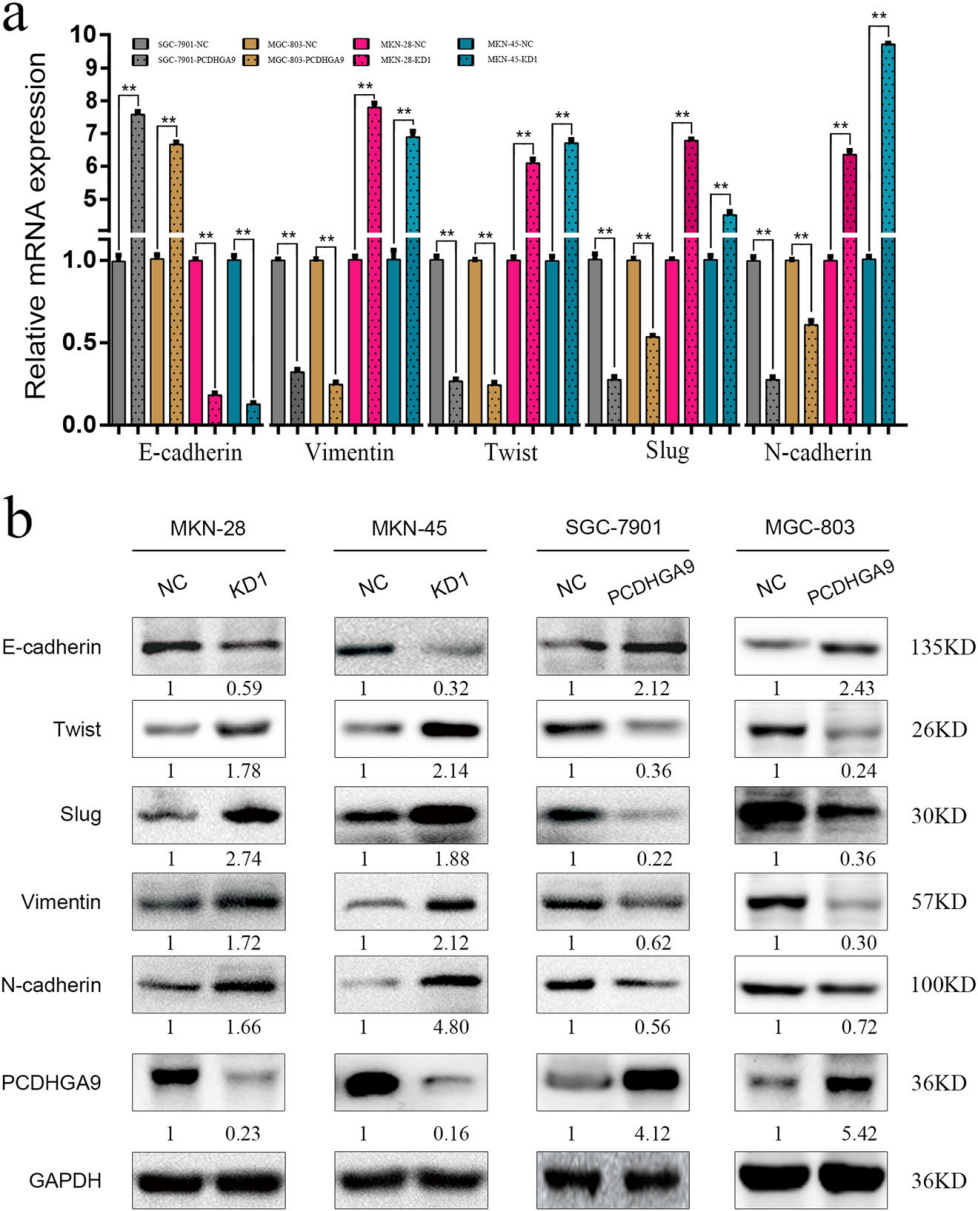
PCDHGA9 inhibits EMT in GC cells. **a**, **b** The expression of EMT markers at mRNA and protein level, including E-cadherin, Vimentin, Twist, Slug and N-cadherin, in PCDHGA9 knockdown or overexpression cells compared with those in the control group was detected by qPCR and Western blot analysis. GAPDH was used to normalize protein expression. The data are presented as the mean ± SEM of three independent experiments. **p < 0.01 by two-tailed Student’s *t*-test.

We previously found that PCDHGA9 expression was associated with EMT-related markers in clinical GC specimens. Thus, we explored whether EMT was involved in the PCDHGA9-mediated alterations in cell motility. Upregulation of PCDHGA9 reduced Vimentin, Twist, Slug and N-cadherin expression and increased E-cadherin expression in SGC-7901 and MGC-803 cells at both the mRNA and protein levels (Fig. 3a, b). Conversely, knockdown of PCDHGA9 in MKN-28 and MKN-45 cells induced Vimentin, Twist, Slug and N-cadherin expression and simultaneously decreased E-cadherin expression (Fig. 3a, b). These results indicate that PCDHGA9 suppresses the EMT process.

### PCDHGA9 suppresses the proliferation and metastasis of GC cells in vivo

**Fig. 4:**
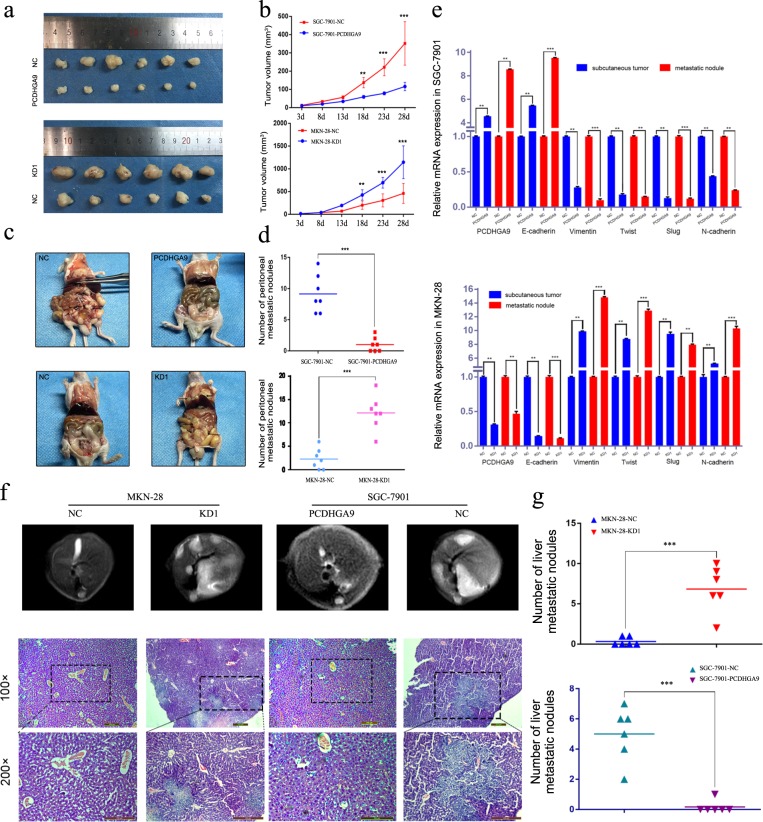
PCDHGA9 supresses GC cell proliferation and metastasis in vivo. **a** The volumes of subcutaneous tumour xenografts of cells were remarkably larger in stable PCDHGA9 knockdown or much smaller in overexpression than their respective control group (*n* = 6 for each group). **b** The graph indicates the tumour volumes, and the xenograft volumes were measured every 5 days. **c** Representative microscopy photographs of peritoneal dissemination in nude mice (*n* = 6 for each group). **d** The numbers of peritoneal dissemination nodules were significantly lower and higher in the PCDHGA9 overexpression and knockdown groups, respectively, than in the respective negative control groups. **e** PCDHGA9 and EMT marker mRNA expression levels in peritoneal dissemination nodules from different groups were detected by qPCR. **f** Representative MRI images and **g** photographs of the HE staining results of liver metastatic nodules. The arrows indicate metastatic nodules. **h** Numbers of liver metastatic nodules in the mice. The data are presented as the mean ± SEM of three independent experiments. **p < 0.01, ***p < 0.001 by two-tailed Student’s *t*-test.

Negative control cells and cells with stable PCDHGA9 overexpression or knockdown were subcutaneously inoculated into nude mice, and tumour growth was measured every 5 days after implantation. Cells with lower PCDHGA9 expression had significantly larger tumour sizes and faster xenograft formation than did the respective control cells (Fig. 4a, b). We next investigated the role of PCDHGA9 in the metastatic phenotype by implanting cells with PCDHGA9 overexpression or knockdown and corresponding control cells into the peritoneal cavities of nude mice. Upon necropsy, we found that mice injected with cells with lower PCDHGA9 expression had more multiple metastatic nodules (Fig. 4c, d). Total RNA was extracted from subcutaneous tumours and metastatic nodules, and the mRNA levels of EMT-related markers (E-cadherin, Vimentin, Twist, Slug and N-cadherin) were evaluated. The results showed that silencing PCDHGA9 induced the expression of mesenchymal mRNAs (Fig. 4e).

In addition, we assessed the effect of PCDHGA9 expression on liver metastasis by injecting modified MKN-28 and SGC-7901 cells into the spleens of nude mice. All mice in the MKN-28-KD1 group (6/6) showed liver metastasis, but fewer mice in the control group had liver metastasis (2/6). Furthermore, PCDHGA9 upregulation in SGC-7901 cells reduced the number of liver metastatic nodules in mice. Representative MRI and HE staining images of the livers from different groups are shown in Fig. 4f, g. Taken together, these results support those from the in vitro experiments, suggesting that PCDHGA9 suppresses GC metastasis in vivo.

### PCDHGA9 inhibits the Wnt/β-catenin signalling pathway in GC cells

**Fig. 5:**
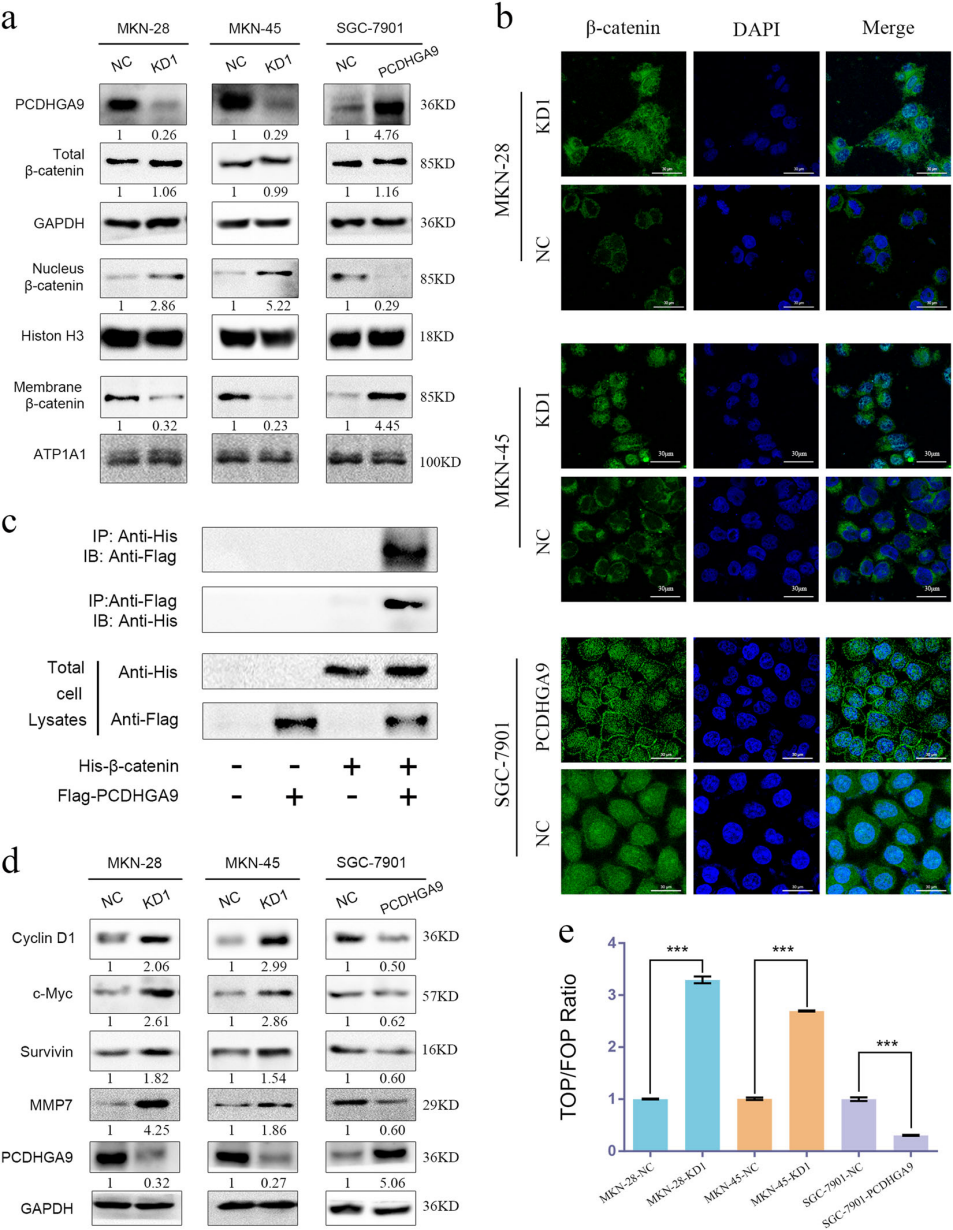
PCDHGA9 negatively regulates the Wnt signalling pathway by modulating β-catenin transcriptional activity in GC cells. **a** Using western blotting, the cellular localization of β-catenin was observed in cells with PCDHGA9 overexpression and knockdown and compared with that in the respective negative control group cells. ATP1A1, histone H3 and GAPDH were used to normalize the protein loading in the membrane, nuclear and total lysate fractions, respectively. **b** Subcellular localization of β-catenin in the indicated cells was detected by immunofluorescence assay. **c** Exogenous co-IP experiments using 293 T cells showed that PCDHGA9 and β-catenin could reciprocally co-immunoprecipitate. **d** The expression of Wnt/β-catenin pathway downstream target molecules (cyclin D1, c-Myc, survivin, and MMP7) was assessed via western blotting. GAPDH was used as an internal control. **e** The TOP/FOP luciferase activity results suggest that β-catenin/TCF4 transcription activity was activated upon PCDHGA9 knockdown but was suppressed upon PCDHGA9 overexpression. The data are presented as the mean ± SEM of three independent experiments. ***p < 0.001 by two-tailed Student’s *t*-test.

Our previous study presented the hypothesis that PCDHGA9, a membrane protein that interacts with β-catenin, constitutes cell–cell junctions and prevents the dissociation of β-catenin from the membrane and its destruction by the degradation complex. To verify this hypothesis, we blocked or upregulated PCDHGA9 expression in different cells and observed the cellular distribution of β-catenin. After cells were cultured, total protein was extracted to create whole-cell lysate, membrane fractions and nuclear fractions, which were then assessed for β-catenin expression using western blotting. The results suggested that PCDHGA9 increased the membrane localization and decreased the nuclear translocation of β-catenin (Fig. 5a). Second, the subcellular localization of β-catenin detected by immunofluorescence analysis further supported our hypothesis (Fig. 5b). Third, an exogenous co-IP assay was performed to validate the hypothesis that PCDHGA9 interacts directly with β-catenin (Fig. 5c). We also explored whether PCDHGA9 exerts its effects by suppressing the Wnt/β-catenin pathway and observed that MMP7, c-Myc, cyclin D1 and survivin, all target genes of the Wnt/β-catenin pathway, were up or downregulated when PCDHGA9 was silenced or overexpressed (Fig. 5d), indicating that PCDHGA9 might inhibit Wnt/β-catenin pathway activation. Moreover, the TOP flash and FOP flash luciferase reporter results suggested that transactivation of the TCF reporter was suppressed by PCDHGA9 overexpression (Fig. 5e); conversely, PCDHGA9 depletion significantly augmented transactivation of the TCF reporter (Fig. 5e). Thus, these data indicate that PCDHGA9 inhibits β-catenin transcriptional activity by directly interacting with β-catenin in GC.

### The Wnt/β-catenin pathway is involved in PCDHGA9-mediated EMT and metastasis

**Fig. 6:**
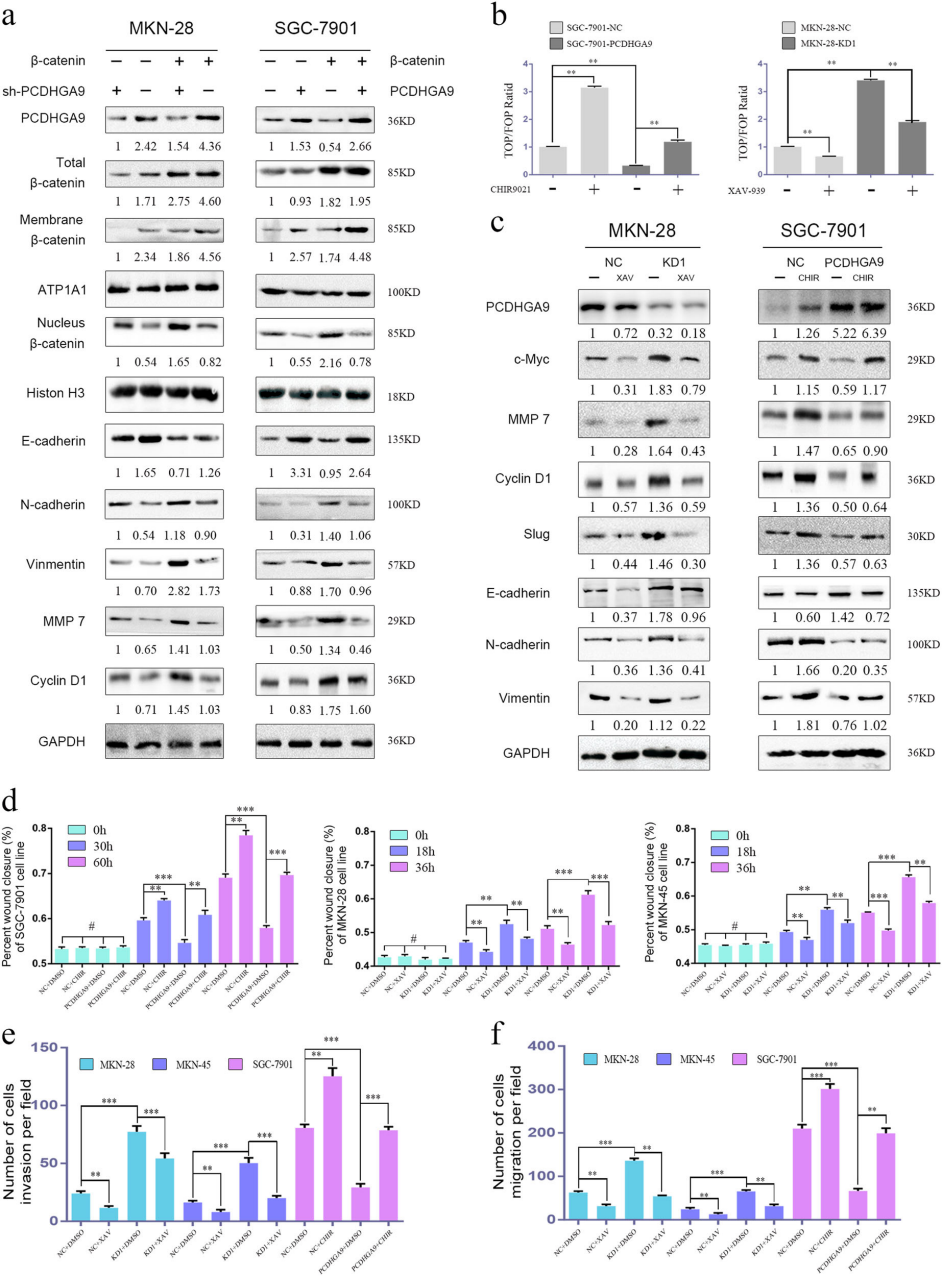
PCDHGA9 inhibits EMT and metastasis via β-catenin redistribution in GC cells. **a** MKN-28 and SGC-7901 cells with stable overexpression or knockdown of PCDHGA9 were transfected with or without β-catenin, and the indicated protein expression was assessed. **b** MKN-45 cells with stable PCDHGA9 knockdown were treated with XAV-939 (10 μmol/l) for 24 h, and SGC-7901 cells with stable PCDHGA9 overexpression were treated with CHIR-99021 (10 μmol/l) for 24 h. Then, the TOP/FOP luciferase activity assay was employed, and **c** the indicated proteins were assessed via western blotting. SGC-7901, MKN-28 and MKN-45 cells with stable overexpression or knockdown of PCDHGA9 were treated with CHIR-99021 (10 μmol/l) or XAV-939 (10 μmol/l) for 24 h as indicated and subjected to a **d** wound healing assay, **e** invasion assay and **f** migration assay. The data are presented as the mean ± SEM of three independent experiments. **p < 0.01, ***p < 0.001 by two-tailed Student’s *t*-test.

β-Catenin plays a significant role in EMT and cancer metastasis and is the central protein of the Wnt signalling pathway^20^. To further investigate whether PCDHGA9 exerts its effects during GC metastasis in the context of the Wnt/β-catenin pathway, we introduced β-catenin into the PCDHGA9-silenced MKN-28 and PCDHGA9-overexpressing SGC-7901 stable cell lines. As shown in Fig. 6a, total β-catenin and nuclear β-catenin levels increased after β-catenin transfection, and blocking PCDHGA9 expression obviously enhanced the nuclear accumulation of β-catenin, which is indicative of EMT induction and Wnt/β-catenin pathway activation in GC cells. Conversely, upregulation of PCDHGA9 significantly reduced the effects of ectopic β-catenin expression.

The TOP/FOP ratio was increased when CHIR-99021, a specific activator of Wnt/β-catenin signalling, was employed, whereas PCDHGA9 overexpression weakened this effect (Fig. 6b). By contrast, XAV-939, a specific inhibitor of Wnt/β-catenin signalling, decreased the TOP/FOP ratio; however, silencing PCDHGA9 reversed this effect. Furthermore, EMT markers and the downstream proteins of the Wnt/β-catenin pathway were assessed by western blotting to verify that PCDHGA9 could suppress Wnt/β-catenin pathway activation and EMT (Fig. 6c). In summary, PCDHGA9 operates by inhibiting the Wnt/β-catenin pathway. For further confirmation, we conducted wound healing and transwell assays using cells cultured in the presence or absence of wither CHIR-99021 or XAV-939 and cells with stable changes in expression (Fig. 6d-f). These results indicate that PCDHGA9 inhibits GC cell migration and invasion by suppressing the Wnt/β-catenin pathway.

### The effects of the canonical Wnt and TGF-β pathways in EMT are synergistic

Under homeostatic conditions, most β-catenin and cadherin molecules are localized to the membrane as an adhesion complex^21^. TGF-β activates EMT by disassociating such complexes to release β-catenin^22^. The Wnt/β-catenin pathway protects dissociated β-catenin from degradation in the cytoplasm^23^. Nevertheless, the cadherin/β-catenin complex provides a powerful force for cell–cell adhesion^24^.

**Fig. 7:**
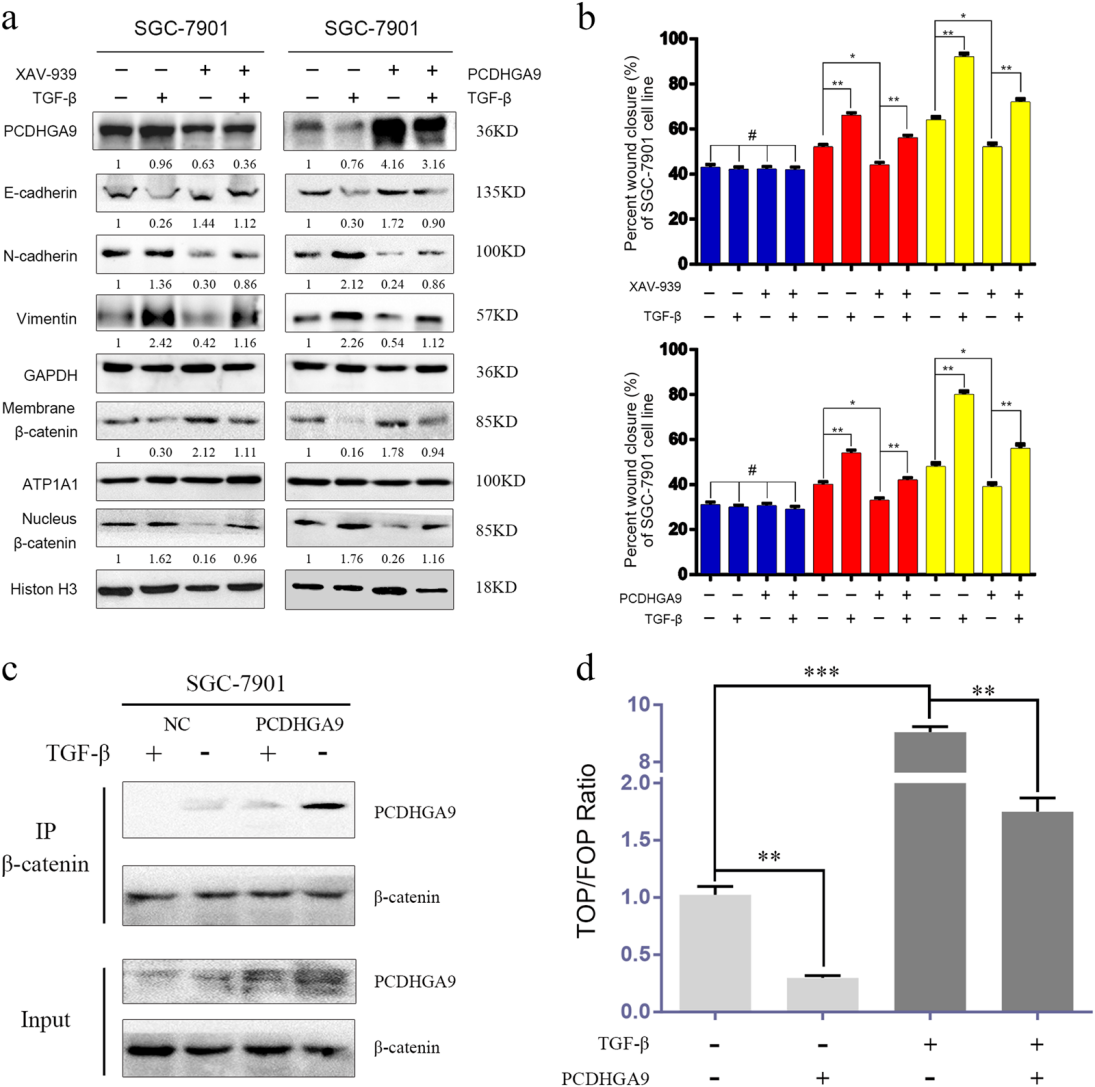
PCDHGA9 interacts with β-catenin and antagonizes the Wnt pathway to attenuate TGF-β-induced EMT. **a** Nuclear translocation of β-catenin and EMT were suppressed by PCDHGA9 overexpression or XAV-939 treatment, as detected by western blotting. Cells were treated with 10 ng/ml TGF-β1 for 48 h. **b** The positive effects of TGF-β1 on migration and invasion were suppressed by PCDHGA9 overexpression or XAV-939 treatment. **c** Immunoprecipitation of β-catenin with PCDHGA9 in PCDHGA9-overexpressing SGC-7901 and negative control cells as detected by western blot analysis. Cells were treated with 10 ng/ml TGF-β1 for 48 h. **d** TOP/FOP luciferase activity results suggested that β-catenin/TCF4 transcription activity was activated after TGF-β1 treatment, whereas it was obviously inhibited by PCDHGA9 overexpression. The data are presented as the mean ± SEM of three independent experiments.

To explore the relationships among PCDHGA9, TGF-β and Wnt/β-catenin, we investigated whether PCDHGA9 can interact with β-catenin, reduce the effect of TGF-β, block activation of the Wnt/β-catenin pathway and attenuate EMT. SGC-7901 cells were treated with TGF-β1 for 48 h, thus activating EMT, as evidenced by increased nuclear β-catenin levels, upregulation of Vimentin and N-cadherin expression, and downregulation of E-cadherin expression. However, using XAV-939 to inhibit the Wnt pathway significantly weakened the effects caused by TGF-β1 (Fig. 7a, left panel). Moreover, a similar result was observed when PCDHGA9 was transfected (Fig. 7a, right panel). Importantly, the increase in cell migration induced by TGF-β1 could be abolished by overexpression of either XAV-939 or PCDHGA9 (Fig. 7b). Furthermore, a decrease in the interaction between PCDHGA9 and β-catenin was observed in response to TGF-β1 treatment. However, ectopic PCDHGA9 expression significantly strengthened the interaction between PCDHGA9 and β-catenin (Fig. 7c). Moreover, PCDHGA9 overexpression was sufficient to prevent TGF-β1-mediated activation of TCF/LEF transcription (Fig. 7d). Taken together, these data suggest that PCDHGA9 combines with β-catenin and antagonizes the Wnt pathway to attenuate TGF-β-induced EMT.

### PCDHGA9 is epigenetically silenced by DNA promoter methylation in GC

**Fig. 8:**
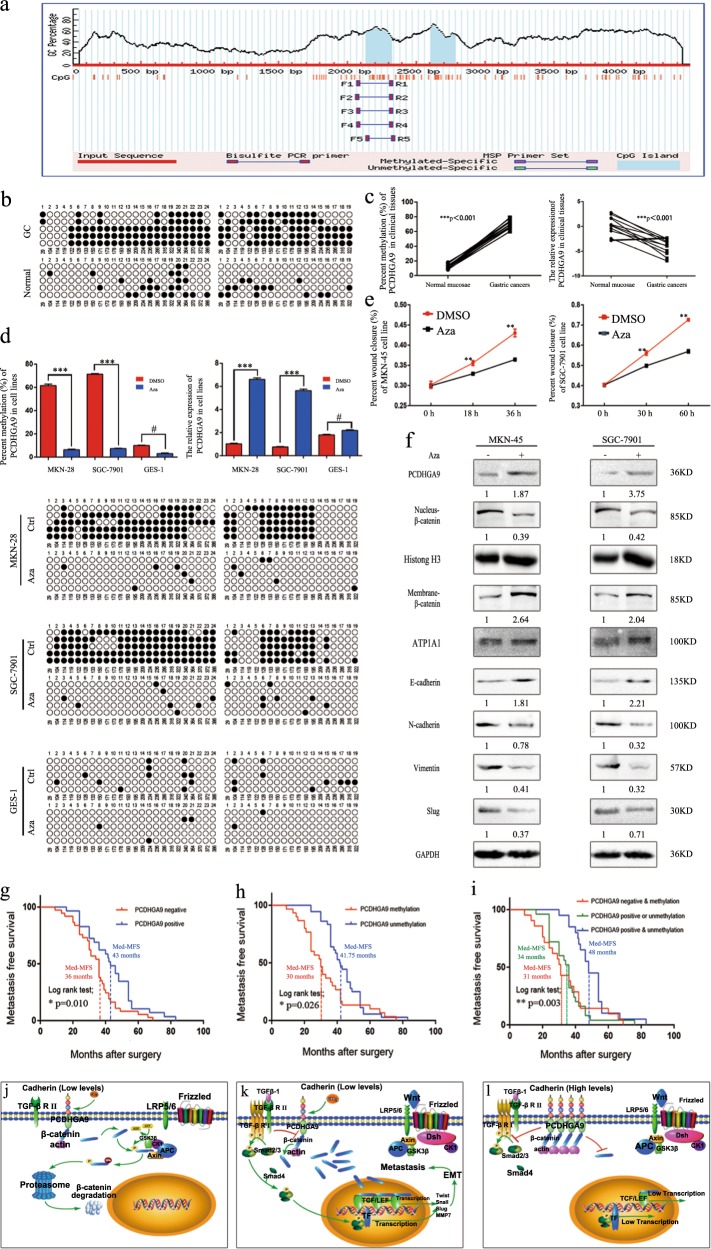
PCDHGA9 is transcriptionally silenced by DNA promoter hypermethylation in GC. **a** Promoter regions of PCDHGA9 with CpG sites indicated by BSP are shown. **b**, **c** BSP revealed that amount of methylation at the PCDHGA9 promoter was much higher in GC tissues than in normal gastric mucosa tissues. **d** Using BSP, we estimated the levels of promoter methylation in MKN-28, SGC-7901 and GES-1 cells and found that treatment with 5-Aza significantly decreased the methylation of the GC cell lines. **e** Demethylation treatment suppressed the migration ability of GC cells, **f** restored PCDHGA9 expression, decreased nuclear β-catenin accumulation and inhibited EMT marker expression. Kaplan-Meier survival analysis (log-rank test) showed that lower expression and higher methylation of PCDHGA9 in GC tissues were correlated with poor prognosis. **g**, **h**, **i** Using Kaplan-Meier survival analysis with log-rank test, compared with patients with lower PCDHGA9 expression, those with higher PCDHGA9 expression had better MFS (*p* = 0.0162), and patients with no methylation had better MFS than those with methylation (*p* = 0.0469). Based on the combination of PCDHGA9 expression and methylation (positive or negative), the patients were divided into three groups. Kaplan-Meier survival analysis (log-rank test) showed that the PCDHGA9-negative and methylated groups had the poorest outcomes, whereas the PCDHGA9-positive and unmethylated groups had the best prognosis (*p* = 0.0004). PCDHGA9 induces GC cell EMT and metastasis in a β-catenin-dependent manner. **j** Methylation of PCDHGA9 and silencing of both the Wnt signalling and TGF-β Smad2/3 pathways. **k** Low PCDHGA9 levels and activation of both the Wnt signalling and TGF-β Smad2/3 pathways. **l** Upregulation of PCDHGA9 inhibits Wnt and TGF-β Smad2/3 pathway activation. The data are presented as the mean ± SEM of three independent experiments.

We found that PCDHGA9 is readily expressed in normal digestive tissues, especially in the stomach (Fig. 8a). As mentioned before, we examined PCDHGA9 expression in normal gastric cell lines and GC cell lines, as well as in matched clinical GC and normal mucosa tissues, and the results showed that PCDHGA9 expression was significantly reduced in GC cell lines and GC tissues. We randomly selected 12 matched pairs of clinical tumours and their corresponding normal mucosae, and BSP demonstrated that PCDHGA9 was methylated in tumour tissues with reduced PCDHGA9 expression but not in normal mucosa samples (Fig. 8b, c). In line with high levels of PCDHGA9 methylation in the MKN-45 and SGC-7901 cells, PCDHGA9 expression in both cell lines was downregulated. Importantly, the methylation and expression of PCDHGA9 could be reversed and restored by treatment with 5-Aza (Fig. 8d). Similarly, treatment with demethylation agents significantly decreased the migration abilities of GC cells (Fig. 8e). We further explored the relationship between PCDHGA9 methylation and EMT by western blotting and found that 5-Aza simultaneously increased the levels of PCDHGA9 and β-catenin in the membrane, decreased the levels of nuclear β-catenin, and reduced the protein expression of mesenchymal markers in GC cells to some degree (Fig. 8f). These results provide evidence that methylation is mediated by the transcriptional silencing of PCDHGA9 in GC.

### There exists a clinical association between PCDHGA9 and metastasis in GC

To further explore the clinical association between the expression and methylation of PCDHGA9 and GC metastasis, we analysed the MFS of 66 GC patients. These patients were divided into two groups based on the median expression of PCDHGA9, and GC patients with high PCDHGA9 expression had a better prognosis and MFS (Fig. 8g). We also divided these patients into two groups according to the median methylation degree of PCDHGA9: methylated group and unmethylated group. Interestingly, patients with methylation of PCDHGA9 had worse outcomes than did patients without methylation (Fig. 8h). Finally, we separated the patients into three groups based on the combined expression and methylation status of PCDHGA9. Patients with unmethylated and positive expression of PCDHGA9 had the best prognosis and MFS. By contrast, patients with methylated and negative expression of PCDHGA9 had the worst MFS and prognosis (Fig. 8i). Taken together, these results suggest that the expression and methylation of PCDHGA9 are associated with metastasis and survival in GC.

## Discussion

In this study, we proposed that the membrane protein PCDHGA9 represses the delivery of nuclear signals and suppresses the progression of GC. The results demonstrate that PCDHGA9 interacts directly with β-catenin and reduces the dissociation of β-catenin from the membrane to antagonize the Wnt/β-catenin pathway and inhibit EMT. Moreover, we found that PCDHGA9 is downregulated in GC tissues and GC cell lines and that this downregulation is accompanied by hypermethylation at the PCDHGA9 promoter.

On the basis of the cDNA array results, we identified candidate genes, from which PCDHGA9 was shown to significantly suppress GC cell migration and upregulate N-cadherin and Vimentin expression more than the other genes. The IHC results from the clinical samples revealed that PCDHGA9 expression was lower in GC tissues than in matched normal mucosa samples, and patients with liver metastasis showed significantly lower PCDHGA9 expression than did patients without metastasis. The in vitro assays demonstrated that PCDHGA9 played a negative role in regulating the migration and invasion properties of GC cells. Furthermore, we showed the suppressive effects of PCDHGA9 on the multiple stages of metastatic GC in vivo by employing subcutaneous implantation, peritoneal injection and liver metastasis models.

Tumour invasion and metastasis are complex and multistage processes in which tumour cells lose their cellular junctions and acquire characteristics that permit them to migrate through the matrix layer and invade into the circulation^10^. Numerous studies support the notion that EMT activation induces the sequential cascade of metastasis^25^. Thus, exploring the key factors affecting this biological change is becoming increasingly significant. PCDHGA9 is a member of the cadherin family that is expressed diffusely in normal brain, spleen, colon and gastric tissues but is weakly expressed or silent in GC tissues. Our previous study indicated that PCDHGA9 could induce GC cell autophagy, apoptosis and cell cycle arrest and inhibit EMT via the TGF-β/Smad2/3 pathway. To further explore the relationship between PCDHGA9 and EMT, we evaluated PCDHGA9 expression and the molecular hallmarks of EMT and found a positive correlation between PCDHGA9 and E-cadherin expression but a negative correlation between PCDHGA9 and N-cadherin, Vimentin and Twist expression in clinical GC tissues, which provided convincing evidence that PCDHGA9 inhibits EMT in GC metastasis. Furthermore, PCDHGA9 overexpression in GC cells enhanced E-cadherin expression and reduced N-cadherin, Vimentin, Slug and Twist expression, whereas PCDHGA9 knockdown elicited opposing effects. We also observed similar results in animal models by extracting RNA from xenograft tumours and peritoneal metastatic nodules. Taken together, these findings suggest the potential effects of PCDHGA9 on EMT, which might be responsible for the metastatic and invasive potential of these cells.

In most epithelial tissues, one common connection among cells is the adhesion complex, which is composed of cadherin and β-catenin^26^. Recent research indicates that cadherin downregulation leads to the disruption of cell–cell junctions, which is the crucial step in EMT that contributes to GC metastasis^27^. Specifically, abnormal β-catenin distribution, which is indicative of Wnt/β-catenin pathway activation, is widely observed in GC^28^. β-Catenin translocation from the membrane to the nucleus initiates TCF/LEF transcription^29^, resulting in increases in Snail and Twist expression and decreases in E-cadherin expression, both of which are significantly important for EMT in GC progression^24^. PCDHGA9 is a member of the cadherin family, but its effect on nuclear signal delivery and EMT in GC cells remains unclear. Interestingly, upon exploring the subcellular localization of β-catenin by western blotting and confocal microscopy, we observed increased membrane β-catenin levels and decreased nuclear β-catenin levels in PCDHGA9-overexpressing GC cells, whereas PCDHGA9-silenced cells showed the opposite phenomenon, which may indicate that PCDHGA9 affects β-catenin distribution and strengthens the stability of membrane-bound β-catenin. Using immunoprecipitation and immunoblotting, we further confirmed that PCDHGA9 directly interacted with β-catenin at exogenous levels. The TOP/FOP luciferase activity assay showed that the transcriptional activity of β-catenin/TCF was inhibited by PCDHGA9 overexpression and that the target proteins of the Wnt/β-catenin pathway were suppressed upon ectopic overexpression of PCDHGA9. In addition, we found that ectopic expression of β-catenin upregulated proteins downstream of the Wnt/β-catenin pathway and induced EMT in GC cells, which could be reversed by PCDHGA9 overexpression. We also detected that TCF transcription activation and the changes in both EMT marker expression and GC cell invasion and migration induced by PCDHGA9 overexpression or silencing were reversed by either CHIR-99021 or XAV-939. Therefore, we conclude that PCDHGA9 can block the canonical Wnt pathway to inhibit EMT by interacting with β-catenin and reducing its dissociation.

Gonzalez DM reported the crosstalk of several signalling pathways closely related to EMT^9^; this finding is in line with our previous hypothesis based on the IPA results that there exists some connections between the Wnt/β-catenin and TGF-β signalling pathways in EMT. We elaborated that in response to TGF-β signals from the microenvironment, the cadherin/β-catenin complex disassociates to release β-catenin^30^, and PCDHGA9 overexpression could reduce this effect and consequently suppress EMT in GC. In the present study, after using TGF-β to induce EMT, we observed that the nuclear translocation of β-catenin or induction of EMT could be reversed by XAV-939. Likewise, enhanced PCDHGA9 expression was also capable of reducing the effects of TGF-β. Thus, we conclude that PCDHGA9 suppresses GC metastasis and attenuates EMT induced by TGF-β and Wnt/β-catenin pathway activation. In this study, we found that PCDHGA9 was abundantly expressed in normal gastric mucosae and other digestive organs but silenced in most GC tissues and GC cell lines in accordance with hypermethylation of its promoter. Importantly, treatment with 5-Aza, a DNA methylation inhibitor, restored PCDHGA9 expression in the GC cell line, which indicated that downregulation of PCDHGA9 was partly mediated by DNA promoter methylation. Furthermore, we explored the clinical significance of PCDHGA9 by analysing the clinicopathologic features of 66 GC patients and found that patients with advanced GC had weak PCDHGA9 expression. Low PCDHGA9 expression (*p* = 0.0162) and PCDHGA9 promoter hypermethylation (*p* = 0.0469) were significantly correlated with MFS. Interestingly, compared with either element alone, the combination of PCDHGA9 expression and promoter hypermethylation reached a higher statistical significance in estimating MFS (*p* = 0.004), which may provide individual GC patients with a more precise appraisal of metastatic risk.

## Conclusions

In summary, this study illuminates the significance of PCDHGA9 in the suppression of GC metastasis through a β-catenin-dependent mechanism. PCDHGA9 interacts with β-catenin directly and weakens the effects of TGF-β and the Wnt/β-catenin pathway in the induction of EMT. Promoter hypermethylation and loss of PCDHGA9 expression in GC may indicate an aggressive phenotype and correlate with poor clinical survival. Moreover, demethylation of PCDHGA9 could be an innovative therapeutic target for GC invasion and metastasis. Therefore, the regulatory mechanism of PCDHGA9 expression and specific reagents that modify the methylation status of PCDHGA9 still require our attention.

## Supplementary information

Supplementary Figure Legends

Supplematery Figure 1

Supplematery Figure 2

Supplematery Figure 3

Supplematery Figure 4
