# Supplementary material for: PCDHGA9 represses epithelial-mesenchymal transition and metastatic potential in gastric cancer cells by reducing β-catenin transcriptional activity
Source: Cell Death Dis. 2020 Mar 30;11(3):206. doi: 10.1038/s41419-020-2398-z (PMC7105466; doi:10.1038/s41419-020-2398-z)
Supplement: Supplementary file 1 — Supplementary Figure Legends [file 41419_2020_2398_MOESM1_ESM.docx]

Supplementary Figure 1: The effects in GC proliferation and migration were observed after knocking down these distinct genes. (a) Wound healing, (b c) Celigo image cytometer. (d) Quantitative real-time PCR of EMT markers (E-cadherin, N-cadherin and Vimentin) in the genes knockdown cells compared with the control group. (The data are presented as the mean ± SEM from three independent experiments)

Supplementary Figure 2: The expression levels of PCDHGA9 in human different tissues showed that PCDHGA9 highly expresses in digestive organs especially in stomach, according to the oncomine database.

Supplementary Figure 3: (a) E-cadherin and Vimentin expression in seven different GC cell lines. (b) The PCDHGA9 overexpression effect was confirmed via qPCR. (c) Gene knocked down by lentiviral delivery of PCDHGA9 shRNAs. In the comparison with different cell lines, expression of PCDHGA9 was much lower after silencing by shRNA-1 (KD1) than the other two sequences. KD-1, infected with Lenti-shRNA-1. The data are presented as the mean ± SEM from three independent experiments.

Supplementary Figure 4: The mechanism illustration of PCDHGA9 suppresses EMT and metastasis in GC
