# Supplementary figures and images for: PCDHGA9 represses epithelial-mesenchymal transition and metastatic potential in gastric cancer cells by reducing β-catenin transcriptional activity
Source: Cell Death Dis. 2020 Mar 30;11(3):206. doi: 10.1038/s41419-020-2398-z (PMC7105466; doi:10.1038/s41419-020-2398-z)

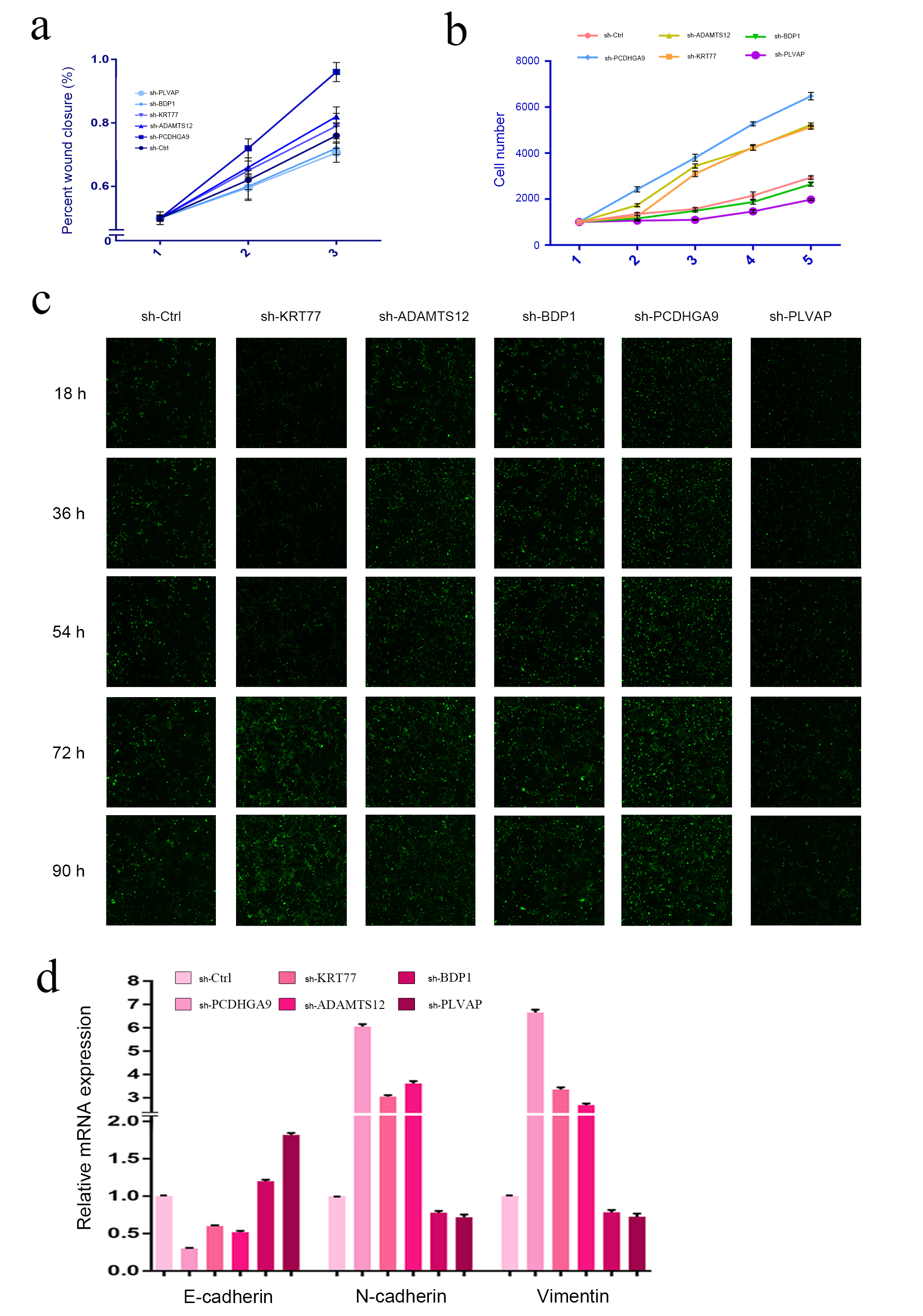

Supplement: Supplementary file 2 — Supplematery Figure 1 [file 41419_2020_2398_MOESM2_ESM.tif]

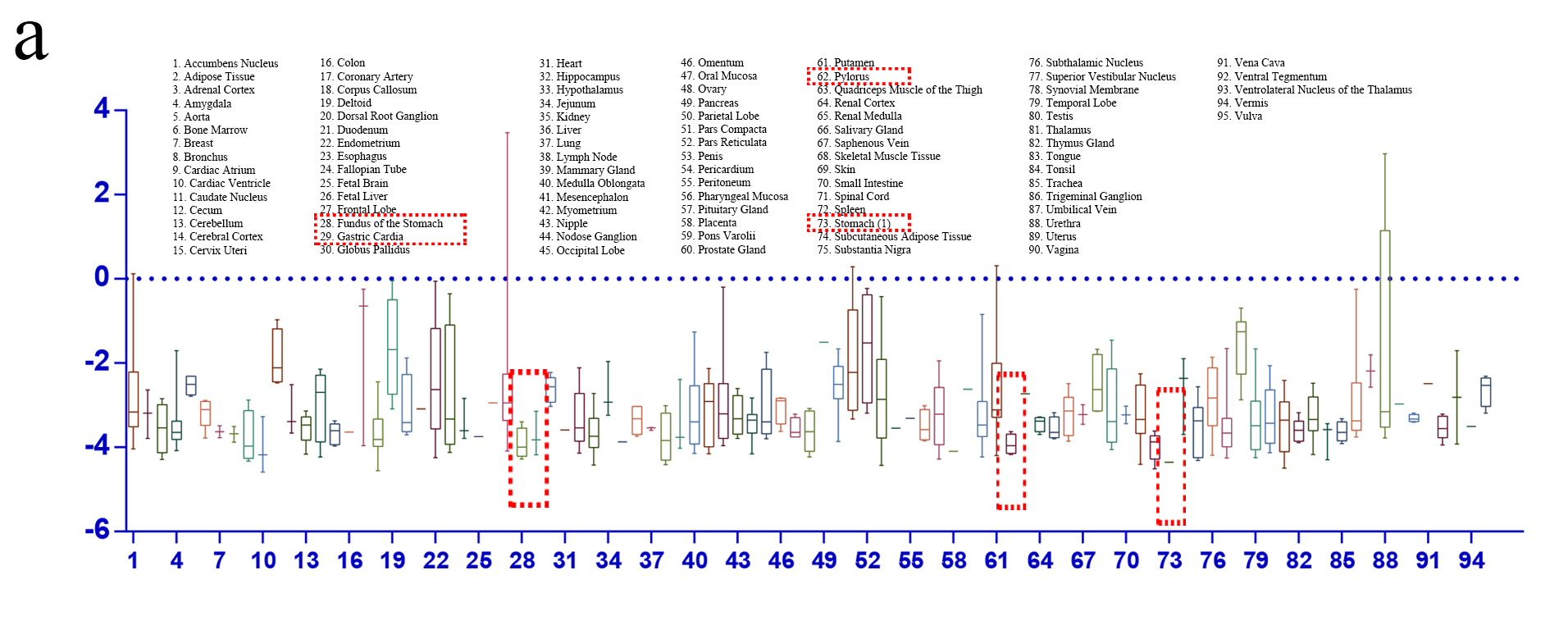

Supplement: Supplementary file 3 — Supplematery Figure 2 [file 41419_2020_2398_MOESM3_ESM.tif]

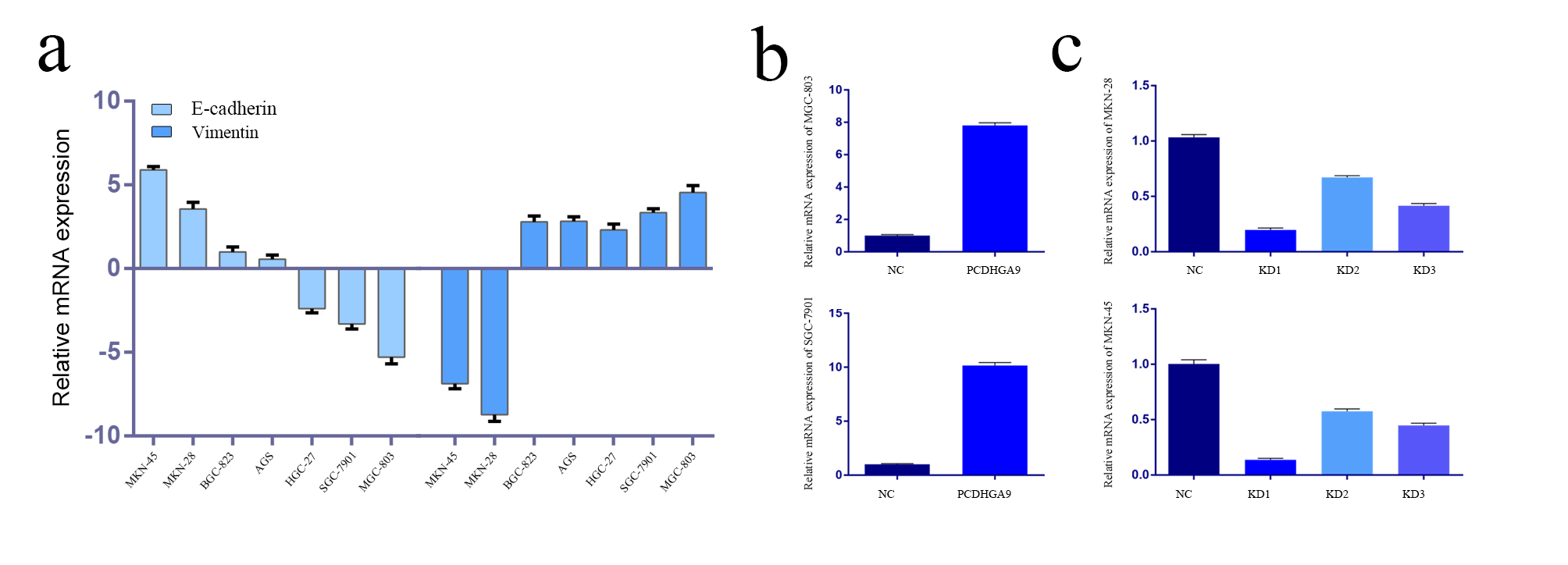

Supplement: Supplementary file 4 — Supplematery Figure 3 [file 41419_2020_2398_MOESM4_ESM.tif]

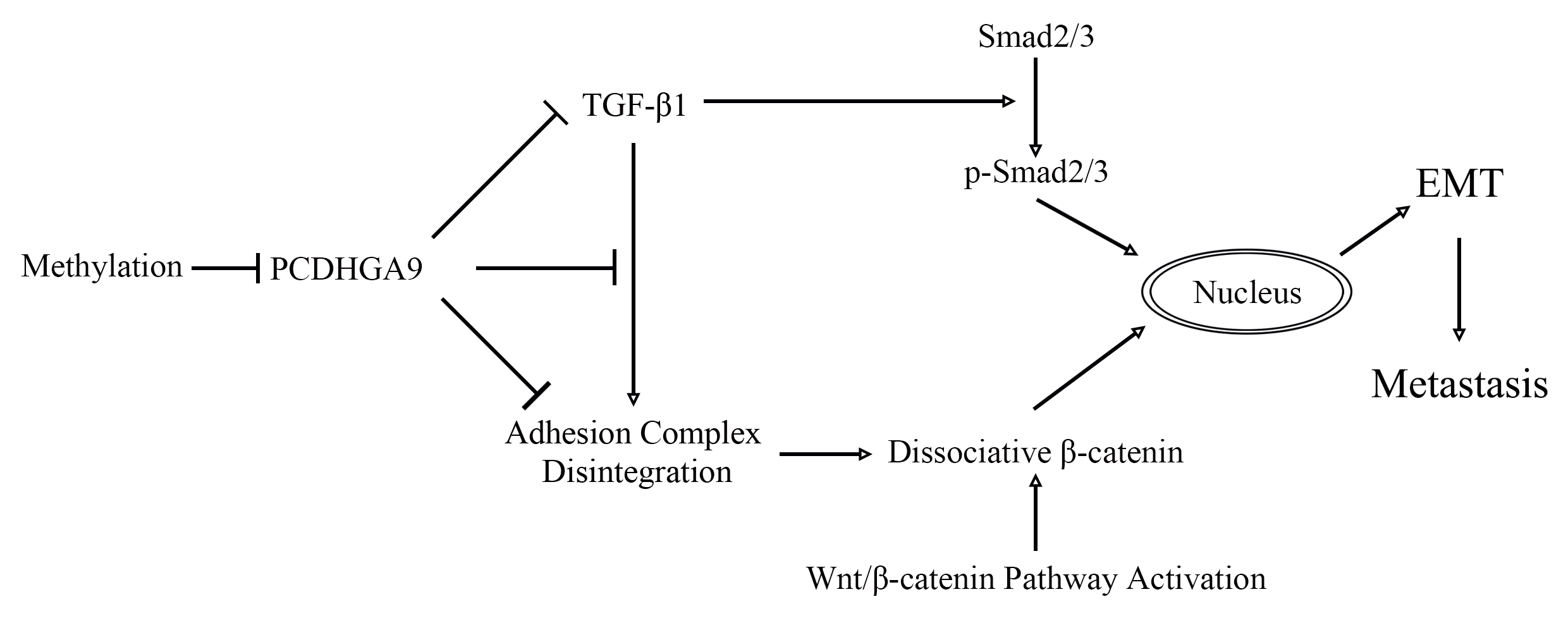

Supplement: Supplementary file 5 — Supplematery Figure 4 [file 41419_2020_2398_MOESM5_ESM.tif]
